# Effect of a Carbohydrate-Rich Diet on Rat Detrusor Smooth Muscle Contractility: An Experimental Study

**DOI:** 10.1155/2017/5796456

**Published:** 2017-10-19

**Authors:** Mustafa Suat Bolat, Sırrı S. Bilge, Ekrem Akdeniz, Onder Cinar, Fatih Firat, Arzu Erdal Agri, Mustafa Bakirtas, Omer Alici, Fikret Erdemir

**Affiliations:** ^1^Department of Urology, Samsun Training and Research Hospital, Health Sciences University, Samsun, Turkey; ^2^Department of Pharmacology, Ondokuz Mayıs University, Samsun, Turkey; ^3^Urology Clinic, Tokat State Hospital, Tokat, Turkey; ^4^Department of Pathology, Samsun Training and Research Hospital, Health Sciences University, Samsun, Turkey; ^5^Department of Pathology, Medical Faculty, Gaziosmanpaşa University, Tokat, Turkey; ^6^Department of Urology, Gaziosmanpaşa University, Tokat, Turkey

## Abstract

**Objectives:**

We aimed to investigate the effect of a carbohydrate-rich diet on detrusor contractility in rats.

**Materials and Methods:**

Sprague-Dawley rats were randomized into two groups. The control group received regular food and water. The study group received carbohydrate-rich diet for six weeks. The rats' detrusor muscle was isolated for pharmacological and histopathological examinations.

**Results:**

In the control and study groups, mean body weights were 431.5 ± 27.6 g and 528.0 ± 36.2 g, respectively (*p* < 0.001). Electrical stimulation of the detrusor strips of the control group resulted in gradual contraction. A decreased contractile response was shown in the study group. Acetylcholine in 10-7-10-3 molar concentration produced a decreased contractile response in the study group, compared to the control group (*p* < 0.01). The study group showed marked subepithelial and intermuscular fibrosis in the bladder.

**Conclusion:**

Carbohydrate-rich diet causes marked subepithelial and extracellular fibrosis and changes in contractility in the detrusor within a six-week period. Changes have higher costs in therapeutic choices and correction of these changes remains difficult. Putting an end to carbohydrate-rich diet would seem to be more cost-effective than dealing with the effects of consuming it in high proportions which should be the national policy worldwide.

## 1. Introduction

An increase in high-energy food intake and the consumption of fat and sugar in foods, along with a sedentary lifestyle, decreased physical activity and lack of exercise, and the development of transportation systems and urbanization all threaten public health around the world. Obesity is a major public health problem affecting populations globally. The worldwide incidence of obesity is increasing significantly. According to the World Health Organization (WHO), 25% of the population are obese and 25% are overweight and prone to obesity [1].

Among the population that falls within the abnormal weight range, 25% are prone to gaining weight genetically. There are more than 400 million obese people and 1.6 billion overweight people living in the world today, and these numbers are predicted to reach 700 million and 2.3 billion, respectively, by 2025. Overweight and obesity are defined as an excessive fat accumulation that presents a risk to health. In recent years, obesity has been declared as one of the four main components of Metabolic Syndrome (MetS) which was first described in 1988 by Reaven [2]. People are considered obese when they have a body mass index (BMI) equal to or above 30. Obesity is known to be associated with many chronic diseases. Morbidity and mortality of the diseases associated with obesity are high. In the urinary tract, renal failure, kidney tumors, kidney stone formation, infertility, and voiding dysfunction have been demonstrated in association with obesity [3–5]. The bladder is the most important organ with regard to normal urination. The lower urinary system is also under risk of obesity [6].

It has been reported that obesity may cause urinary incontinence by affecting other organs and systems including neurogenic diseases that may contribute to pelvic floor and urethral dysfunction, delayed median nerve conduction, and likelihood of lumbar intervertebral disc herniation. That study had some limitations: although neuromuscular dysfunction was mentioned, that study was mainly set up on the basis of obesity and stress urinary incontinence. The lack of isolated pharmacological and histological experiments on the detrusor was another limitation [7].

Micturition occurs involuntarily at the beginning of life. After the age of 3 to 5 years, it is regulated voluntarily. The micturition process has complex pathways at several levels, such as the brain, spinal cord, and peripheral nervous system, and it is mediated by multiple neurotransmitters. The urinary control mechanism is sensitive to various disturbances due to its complexity. The role of obesity in bladder dysfunction has gained importance in recent years. A high-fat diet, as well as obesity, may have negative effects on detrusor functions [8]. The food industry's use of a higher proportion of carbohydrate and the increasing prevalence of obesity may lead to negative effects on detrusor functions.

In this present study, we aimed to investigate the functional and histological effects of a carbohydrate-rich diet on detrusor smooth muscle contractility in rats.

## 2. Materials and Methods

This experimental animal study was approved by the Ondokuz Mayıs University Animal Care and Use Committee and was carried out in 2016. Mature male Sprague-Dawley rats aged 9–12 weeks were obtained from Gaziosmanpaşa University vivarium sources. All procedures and protocols were conducted in accordance with the* Guide for the Care and Use of Laboratory Animals* (NIH publication 865-23, Bethesda, MD, USA). Rats were housed in a temperature- and humidity-controlled room (22°C and 60 ± 5%, resp.) in a 12 h light/dark cycle. Rats were randomized into two groups, each containing 10 animals. The control group rats received regular food and water ad libitum. The study group received a 37–40% fructose diet. Six weeks later, the rats were weighed and killed by cervical dislocation and the detrusor muscle was evaluated.

### 2.1. In Vitro Experiments

The detrusor with the urothelium was sliced into 3 × 10 mm strips. The strips were transferred to organ baths containing 10 ml Krebs Henseleit solution (composition in mM: NaCl 118, KCl 5.6, CaCl_2_ 2.5, MgSO_4_ 1.2, KH_2_PO_4_ 0.9, NaHCO_3_ 25, and glucose 11). The solution was continuously gassed with 95% O_2_ and 5% CO_2_ and maintained at 37.2°C and pH 7.4. Each preparation was treated through a ring electrode (3 mm internal diameter, 1 cm apart) (MLA0305/8, AD Instruments, UK) connected to a stimulator (Grass, USA). The lower end of the preparation was attached to a holder, and the other end was attached to an isometric force transducer (MLT0201, AD Instruments, UK) coupled to a Quad-Bridge amplifier (ML118, AD Instruments, UK) that was connected to a digital recorder (PowerLab/4SP) (AD Instruments, UK). Strips were allowed to equilibrate for 1 hour followed by 1 g of tension. The Krebs Henseleit solution was refreshed every 15 minutes. The functional viability of preparation was assessed by the addition of acetylcholine and electrical field stimulation for the detrusor.

Acetylcholine (Sigma, USA) was administered to assess detrusor smooth muscle contractions in a cumulative manner (10-7-10-3 M) and produced cumulative concentration-response curves. Each incremental concentration was added when the response to the previous concentration reached a plateau and stabilized. The frequency-response curves were constructed as follows: square wave pulses (100 V, 0.5 ms) were delivered for 20 s at increasing frequencies (2–64 Hz) with a 4 min interval between two consecutive frequency steps.

### 2.2. Statistical Analysis

All data are expressed as the mean ± SD. Data analyses were performed using GraphPad Instat software (v. 3.0) (GraphPad, USA). Following the assurance of a normal distribution of data, one-way analysis of variance (ANOVA) with the Tukey-Kramer post hoc test was used for multiple comparison. Values of *p* < 0.05 were regarded as significant.

## 3. **Results**

**Figure 1:**
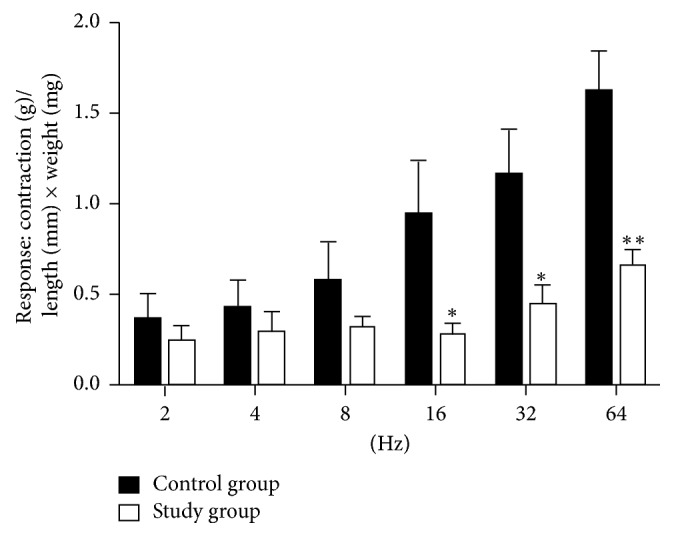
Effects of a fructose-rich diet on electrical field stimulation-evoked contractions; ^*∗*^*p* < 0.05 and ^*∗∗*^*p* < 0.01, compared to the corresponding frequency of control group.

**Figure 2:**
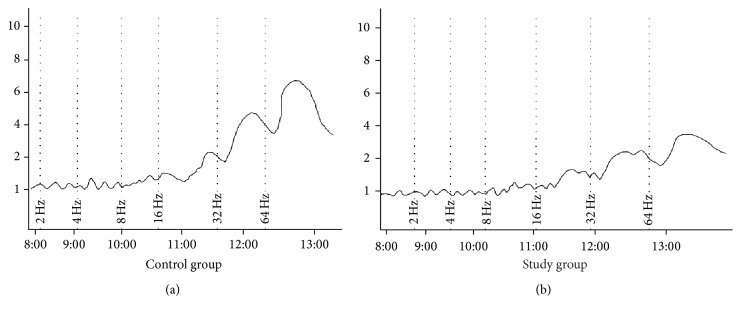
Recordings of responses of electrical field stimulation evoked (100 V, 0.5 ms, 2–64 Hz) in control and study groups.

**Figure 3:**
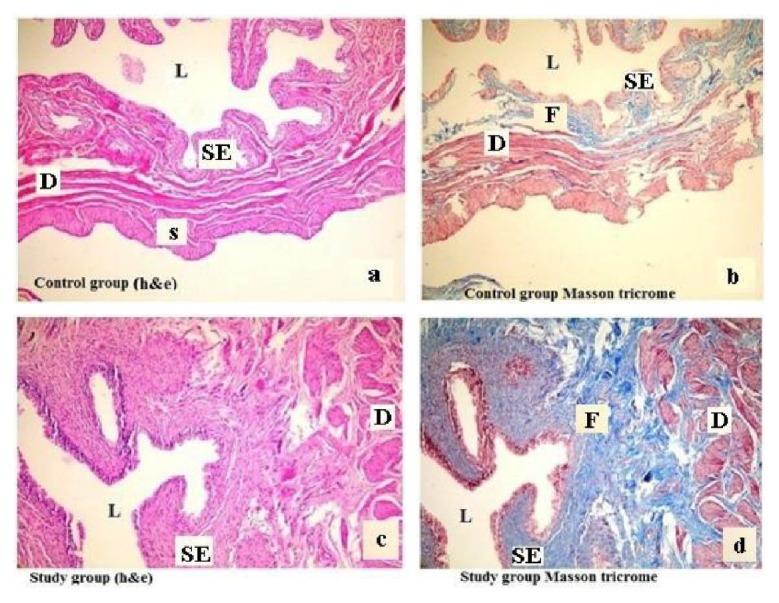
(a, c) Histopathological appearance of the urinary bladder of the groups (H&E stain, 100x). (b, d) Masson trichrome stain in both groups, respectively. L: lumen; SE: subepithelium; D: detrusor; S: serosa. Care must be taken with marked subepithelial and extracellular fibrosis (d) when compared to the control group (b).

**Figure 4:**
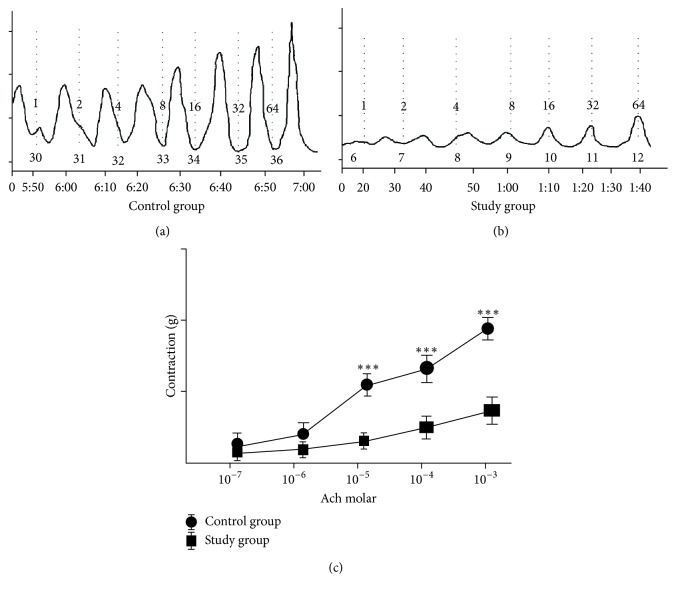
(a, b) Recording of responses to increasing concentrations of acetylcholine (10-7-10-3 in control and study groups). (c) Effects of the fructose-rich diet on acetylcholine-evoked (10-7-10-3 M) contractions; ^*∗∗∗*^*p* < 0.001 compared to the control group.

Control and study groups' weights were recorded at the beginning and end of the study after six weeks. At the beginning of the study, the mean body weights of the control group and study group were 315.1 ± 49.5 g (302–378 g) and 312.5 ± 42.4 g (297–368 g) (*p* = 0.47), respectively. After a six-week period, in the control and the study group, the mean body weights were 431.5 ± 27.6 g (405–476) and 528.0 ± 36.2 g (476–571) and were found to be statistically significant, respectively (*p* < 0.001). Electrical stimulation of the detrusor strips of the control group resulted in a gradual contraction in frequencies between 2 and 64 Hz. The study group fed with a carbohydrate-rich diet revealed significantly decreased contractile responses when compared with the control group in the same frequencies of electrical stimulation (Figure 1). In both groups, with EFS in frequencies of 2, 4, and 8 Hz, there was no difference in detrusor muscle contractions, but 16 Hz (*p* < 0.05), 32 Hz (*p* < 0.05), and 64 Hz (*p* < 0.01) induced diminished contractions in the study group compared to the control group (Figures 2(a) and 2(b)). Urinary bladder specimens were obtained for histopathological examination and stained with hematoxylin and eosin (H&E) (×100) and Masson trichrome stain (×100) in both groups. The microscopic appearance of the detrusor revealed a normal cellular arrangement in the groups (Figures 3(a) and 3(c)), but in the study group, marked subepithelial fibrosis and intermuscular fibrosis were seen with Masson trichrome stain compared to the control group (Figures 3(b) and 3(d)). Acetylcholine (Ach) at 10-7-10-3 molar concentration caused dose-dependent contractions in the smooth detrusor muscle strips in the control group, but no significant dose-dependent response was recorded with increasing concentration of Ach in the study group (*p* < 0.01) (Figures 4(a), 4(b), and 4(c)).

## 4. **Discussion**

The micturition cycle consists of two different processes: filling and storing in the bladder and bladder emptying or voiding. The presence of an adequately coordinated detrusor contraction, lack of infravesical obstruction, and decrease in bladder neck pressure are also needed for normal voiding. Despite an increase in urine volume in the bladder, low intravesical pressure with appropriate sensation, the absence of involuntary detrusor contraction, and closure of the bladder neck are necessary for storage. Relaxation of the bladder neck is coordinated by a noradrenergic-noncholinergic mechanism mediated by nitric oxide. Voiding function is provided by the inhibitory effect of some neurotransmitters, such as glycine, gamma-aminobutyric acid (GABA) opioids, purines, and the noradrenergic system at various levels of the neural axis. Bladder filling and consequent wall distention may also result in the release of factors that may have an influence, such as acetylcholine (Ach), adenosine triphosphate (ATP), nitric oxide, and prostaglandin. Pharmacologically, M1, M2, and M3 receptor subtypes have been found in the human bladder [9]. Stimulation of M3 receptors by acetylcholine leads to smooth muscle contraction [10]. Obesity is a global health problem that affects multiple organs and systems with such conditions as hypertriglyceridemia, type 2 diabetes, hypertension, MetS, coronary heart disease, stroke, cancers, gallstones, female infertility or menstrual irregularities, erectile dysfunction, nonalcoholic fatty liver disease, and osteoarthritis [11–16]. There are a wide variety of etiological causes of obesity, such as lack of energy balance, inactive lifestyle, environment, genes and family history, hormonal disturbances, emotional factors, pregnancy, sleep disorders, and, as a new context, a carbohydrate-rich diet. Since the mid-1970s, many clinical and experimental studies have been carried out using high-fructose corn syrup as used in the food industry. Lee et al. stated that fructose-fed rats within abnormal voiding behavior showed obvious neuromuscular and myogenic alterations and upregulation of M2 and M3 muscarinic receptors [17, 18]. They found those findings following six months of high-fructose corn syrup (HFCS) feeding. Contrary to this, another study advocated that serious fibrosis of the bladder wall and downregulation of the muscarinic M3 receptor led to diminished contractility of the urinary bladder [8]. Discrepancies between studies can be explained by the fact that studies with no reduction in M3 receptors have been conducted on a high-fat diet, but other studies in which M3 receptor downregulation was detected used a fructose-rich diet. Palma et al. showed a correlation between overactive bladder symptoms and BMI among 1050 middle-aged women. They reported a statistically significant difference in terms of nocturia, urgency, and urge incontinence in women with a BMI ≥ 30 compared with women with a BMI < 25 [19]. Similarly, Chang et al. stated that children with a higher BMI had higher urgency symptoms [6]. Another population-representative, cross-sectional, Internet-based survey from the United States, the United Kingdom, and Sweden, which was part of EpiLUTS, showed a relationship between obesity and urinary incontinence subtypes, such as stress incontinence, urge incontinence, overflow incontinence, or mixed incontinence, suggesting different mechanisms of incontinence other than a purely mechanical stress subtype. Among the patients, obesity rates were found to be the highest among mixed incontinence irrespective of gender and stress incontinence with overactive incontinence in women and urge incontinence with overactive incontinence in men [20]. Several mechanisms may play a role in bladder functions. Obesity may cause increased intra-abdominal pressures and this could adversely stress the pelvic floor and may contribute to genuine stress incontinence. Other possible mechanisms may be due to impaired neuromuscular function (urge incontinence) [7]. Systemic oxidative stress is thought to lead to vascular damage in the detrusor and urinary sphincter. Excessive urine production associated with diabetes may also lead to urge incontinence [21]. The effects of a carbohydrate-rich diet were pharmacologically and histologically investigated in this present study. HFCS was first introduced to the food industry in the 1970s and grew in the mid-90s [22]. It has been reported that countries using HFCS in their food supply have a 20% higher prevalence of diabetes, regardless of total sugar intake and obesity levels. Fructose is metabolized differently from glucose, and its metabolism occurs independently of insulin, primarily in the liver [23]. At the end of the metabolism, it may be readily converted to fat. Our results showed that rats fed with HFCS gained 23.8% more body weight compared with the control group. Weight gain can be explained with the noninsulin dependence of fructose in metabolism.

In our study, HFCS was shown to cause marked subepithelial fibrosis within a six-week period and fructose had dual negative effects on detrusor function, including neuromuscular and histopathological aspects. Our opinion is that the lack of flexibility of the fibrotic tissue could adversely affect the neuromuscular mechanism and this could affect voiding behavior. A lack of cystometric evaluation is a limitation of our study. Another limitation is the lack of weekly weight monitoring of the groups. Obesity and marked subepithelial fibrosis are the most significant findings of this study. Oberbach et al. reported that weight loss had no impact on regression of the bladder fibrosis in their experimental study [24], but some advocated that a decrease in BMI caused relief for lower urinary tract symptoms [25].

## 5. Conclusion

It is obvious that HFCS causes marked subepithelial and detrusor fibrosis and this may cause negative effects on detrusor function and metabolic pathways within a six-week period. Correction of these changes remains difficult and has higher costs in therapeutic choices. Our aim is to create public awareness of high-fructose-caused obesity. Putting an end to fructose-rich diets would seem to be more cost-effective than dealing with the effects of consuming it in high proportions and this should be the national policy worldwide.
